# Supplementary figures and images for: A pre-training and self-training approach for biomedical named entity recognition
Source: PLoS One. 2021 Feb 9;16(2):e0246310. doi: 10.1371/journal.pone.0246310 (PMC7872256; doi:10.1371/journal.pone.0246310)

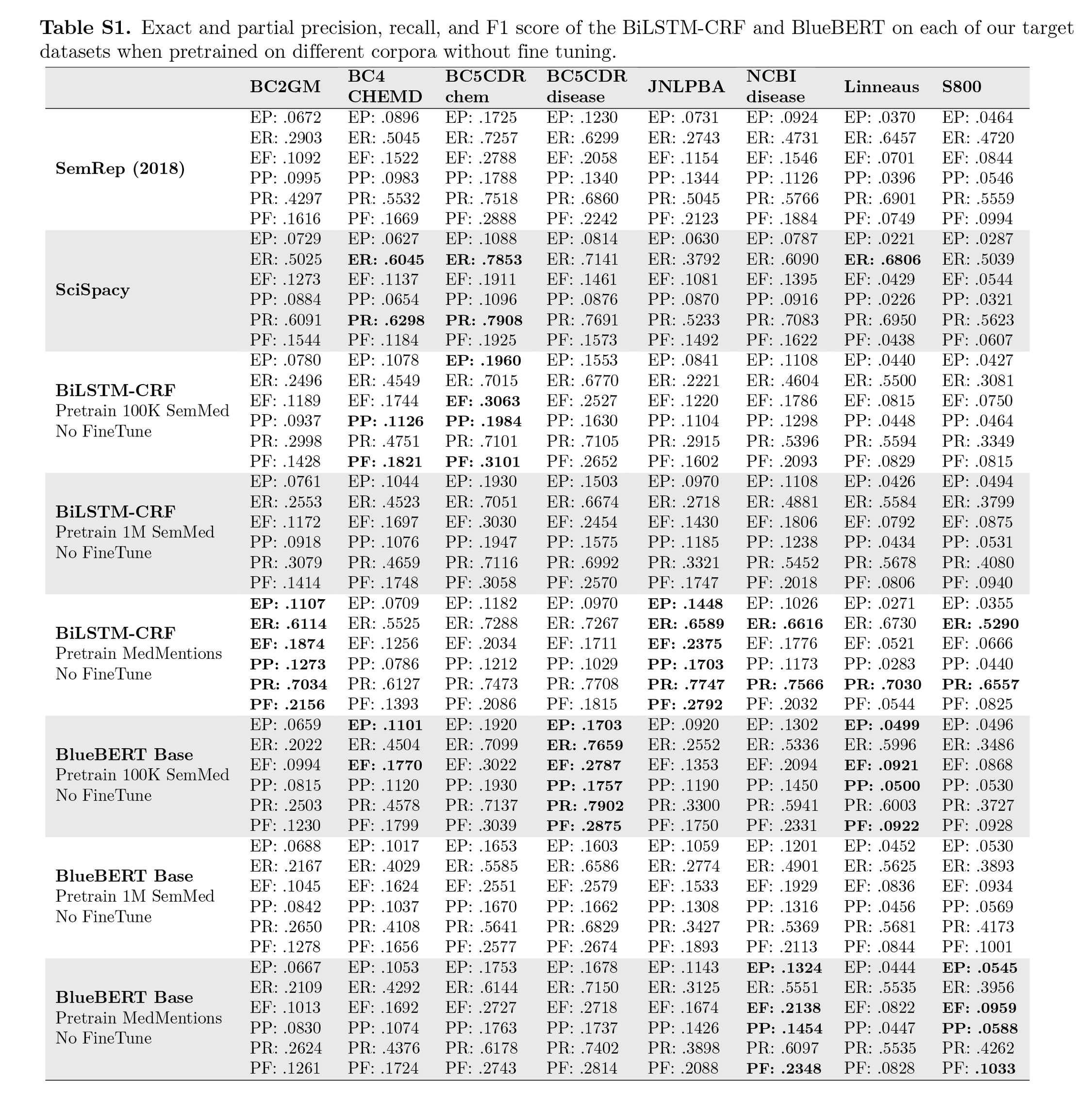

Supplement: S1 Table — (TIF) [file pone.0246310.s001.tif]

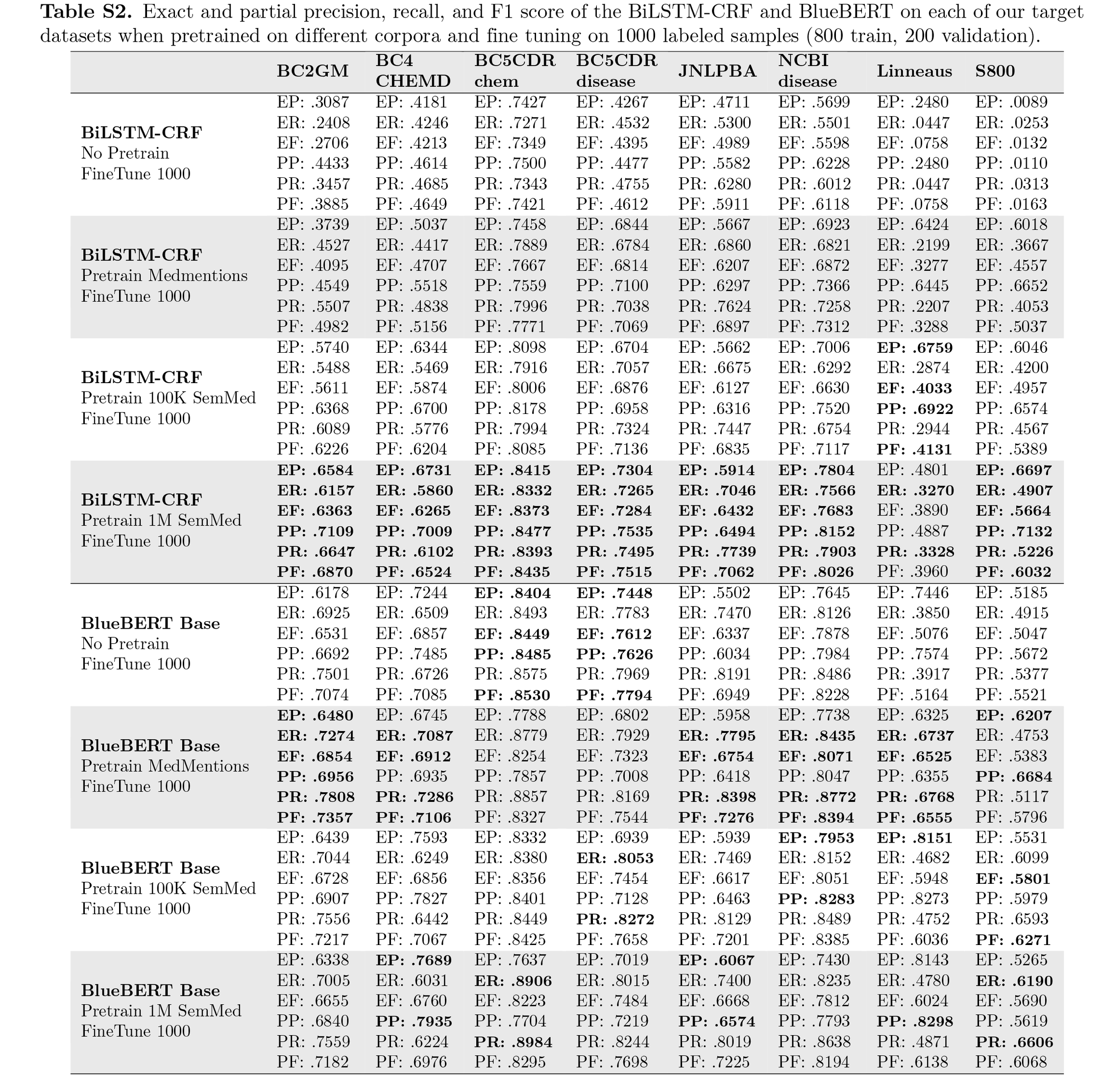

Supplement: S2 Table — (TIF) [file pone.0246310.s002.tif]

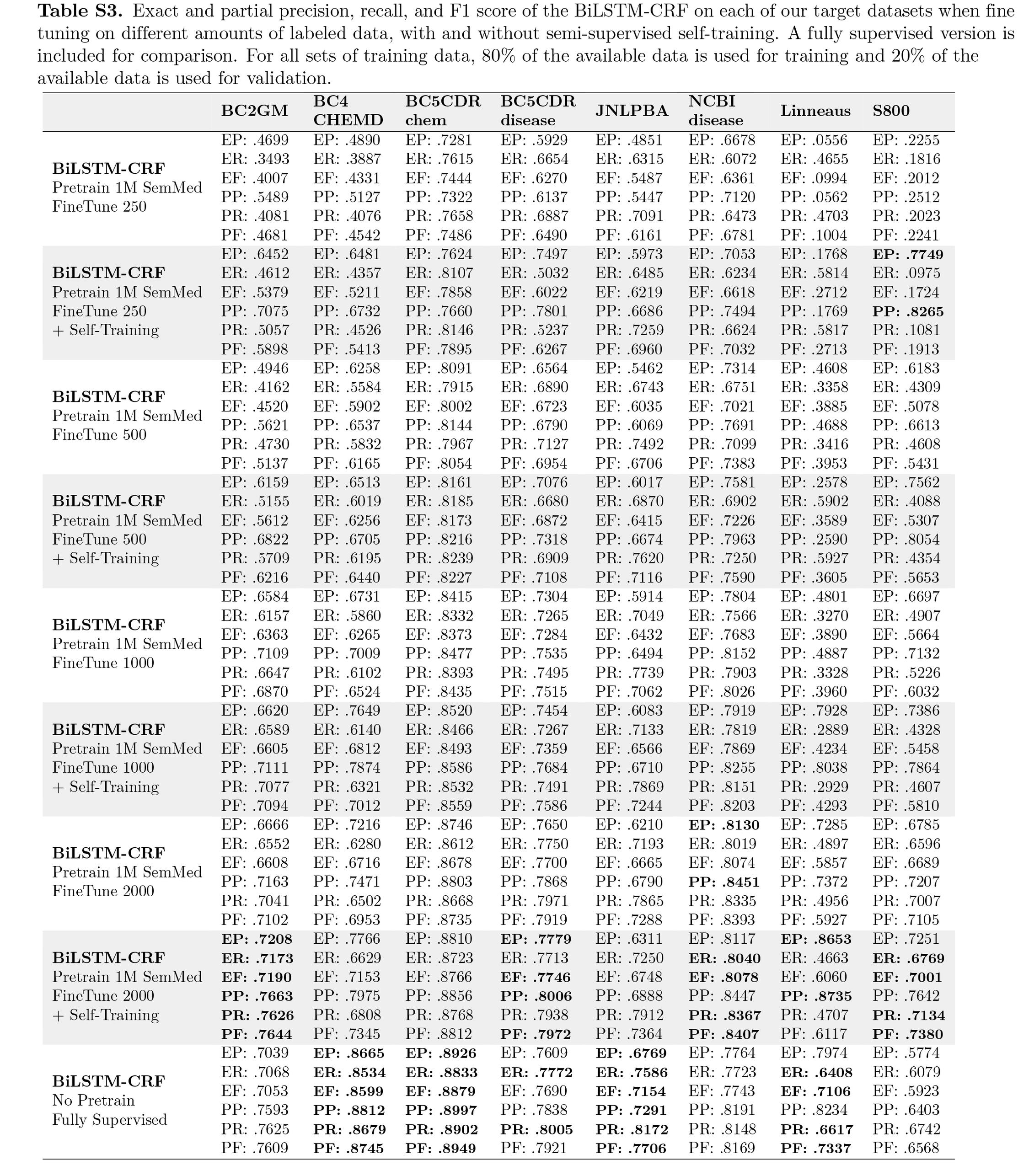

Supplement: S3 Table — For all sets of training data, 80% of the available data is used for training and 20% of the available data is used for validation. (TIF) [file pone.0246310.s003.tif]

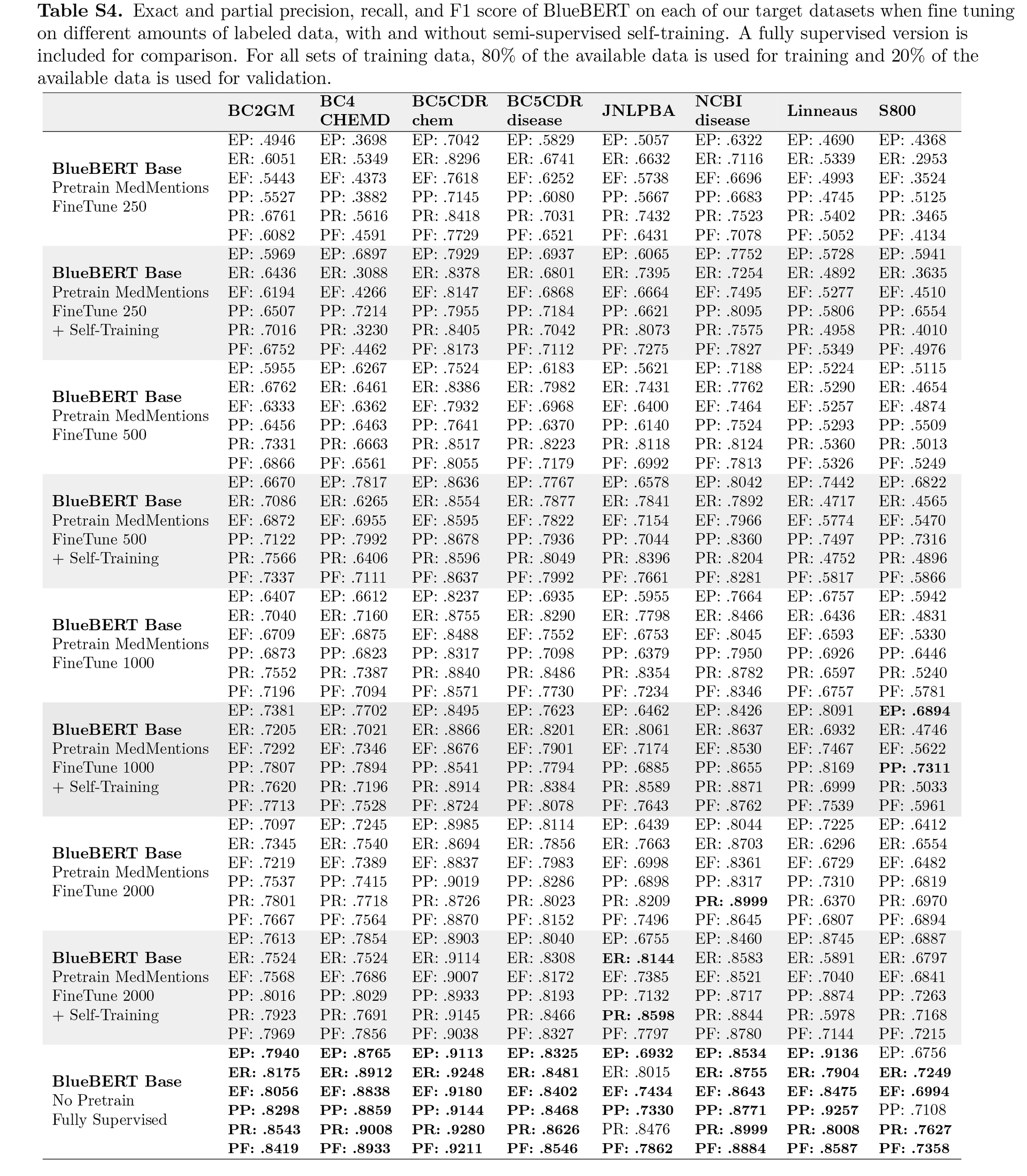

Supplement: S4 Table — For all sets of training data, 80% of the available data is used for training and 20% of the available data is used for validation. (TIF) [file pone.0246310.s004.tif]
