# Supplementary material for: A pre-training and self-training approach for biomedical named entity recognition
Source: PLoS One. 2021 Feb 9;16(2):e0246310. doi: 10.1371/journal.pone.0246310 (PMC7872256; doi:10.1371/journal.pone.0246310)
Supplement: Attachment — Submitted filename: reviewer_response.docx [file pone.0246310.s005.docx]

We would like to thank our reviewers for their thoughtful and insightful comments. This feedback has been extremely helpful in identifying limitations of our work and has enabled us to include additional relevant experiments and improve the overall quality of our paper. We have addressed each reviewer’s comments to the best of our ability below.

Response to Reviewer 1 Comments

**Reviewer-1**

This paper investigated cumulative effect of pre-training and semi-supervised self-training on multiple datasets. The pre-training was executed on three datasets, SemMed 100k, SemMed 1M, and MedMentions. The authors showed that the self-training method with the pre-trained parameters can boost the performance. Although the performance improvement by pre-training and semi-supervised self-training was previously known in the NER task, this paper has contribution on the cumulative effect of pre-training and semi-supervised self-training in low resource settings. However, this paper constructed a low resource setting by reducing the number of training samples from a large size of corpus. Thus, it is important to evaluate the proposed approach to the real application with a small number of NER annotation.

Major:
**Point 1:** The proposed approach was tested on the applications with a large number of NER datasets such as disease names, chemicals, and genes. Because UMLS semantic types, which were used for pre-training, include entities such as chemicals and phenomenon or process, pre-training datasets already included many similar entities. The authors need to present the domain similarity between the tested dataset and pre-training datasets to make sure that the experiment settings are real low resource settings.

**Response 1:**

We agree with the reviewer that there may be significant overlap between the entity types in our pre-training datasets and the downstream NER test sets. Therefore, our claim that these datasets are “low-resource” because we limit the number of labelled training examples may not be appropriate. To address this, we have made the following changes:

1. We have reframed our main claims to focus on NER applications with limited training samples that focus on common biomedical entities such as those covered in UMLS. We have also adjusted our title to reflect this change. We note that because UMLS covers a very wide range of biomedical entities, we expect that most biomedical NER applications will have some overlap with UMLS, which is why our pre-training datasets are so appropriate.
2. We have added a new Table 3 on page 12 in which we measure the percentage of unique entities in each downstream NER dataset that also appear at least once as labeled entities in the pre-training datasets. We further show in our results section that there is no clear relationship between the precent overlap in entities and how much pre-training improves performance on the downstream NER dataset. Low entity overlap in the pre-training dataset does not necessarily mean that transfer learning will not give a significant performance boost, and vice versa.
3. In our Discussion section (page 22 and 23), we have included an additional set of experiments on TAC SRIE, which we consider a more accurate representation of a low-resource dataset. The TAC SRIE dataset consists of the "Material and Methods" section from 100 toxicology papers, and the entity types of interest are generally not covered within the entity types from UMLS. We further note that we include a sub-experiment in which we test on TAC SRIE without the “species” and “sex” entity types -- we exclude "species" because this entity type is most likely to overlap with UMLS and therefore the pre-training sets, and we exclude "sex” because this entity type is usually a simple keyword search for "male" or "female”. We show that pre-training and self-training can still give performance boosts on this dataset. However, we state that a broader study utilizing a wider range of different types of low-resource NER datasets is required to establish the effectiveness of our methods in low-resource settings.

**Point 2:** The authors mentioned that CRISPR and Covid-19 are examples of the application areas with a low resource. Thus, it is important to test the proposed approach using a real low resource data set, where the size of a corpus set is small and similar pre-training data sets are not available.

**Response 2:**

We agree with the reviewer, and we have added an additional experiment on the TAC SRIE dataset (see point 3 in response 1).

We note that we have removed any mentions to CRISPR and COVID from our paper because we do not include any experiments directly related to these subjects.

**Point 3:** What was the criterion to set the confidence threshold to 99.75%? When set to lower threshold, the model is likely to propagate errors more than the knowledge. How can we find the proper threshold?

**Response 3:**

This is an excellent question. We have included a new set of experiments in our Discussion section (please see page 21) about how the choice of confidence threshold affects precision and recall when it comes to self-training. We note three general trends: (1) First, lower confidence thresholds require fewer iterations of self-training because each iteration adds more samples from the unlabeled set and therefore samples from the unlabeled set are used up more quickly. We noticed that for some datasets, extremely high thresholds also require fewer iterations of self-training because after a number of initial iterations, no more samples from the unlabeled set make it pass the threshold. (2) Second, too low or too high of a confidence threshold results in lower performance in terms of overall F-score; the optimal range for the confidence threshold varies by dataset. Finally, (3) the specific behavior of how different confidence thresholds affect precision, recall, and F-score is dependent on the dataset and model.

Ultimately, it is up to the user to tune the confidence threshold as a hyperparameter based off the needs of their experiment, as the optimal confidence threshold varies based on the dataset and model used.

Minor
**Point 4:** Please check the grammar errors. For examples, "We note the this", "NER ragging", "naturaly language processing" and so on.

**Response 4:**

Thank you for pointing these out. We have gone through our manuscript and have corrected these and other grammatical errors.

Response to Reviewer 2 Comments

**Reviewer-2**

I would like to thank the authors for this well-written and easy to read article. The article proposes that transfer learning and self-training can be beneficial for biomedical NER tasks with small training sets. Through various ablation studies they show the benefit of transfer learning and self-training individually and also delve into some of the unexpected results in the Discussion. I really like the paper, but unfortunately don’t feel that the paper’s conclusions on “low-resource settings” are warranted. While I accept that pre-training and self-training improve performance, there needs to be better comparisons to show it in context with other methods. I describe my concerns below.

Major
**Point 1:** I don’t accept that the datasets used for testing and the methods for down sampling them are actually low-resource settings. The entity data used for pre-training is all based on UMLS terms which are a superset of all the downstream datasets evaluated. A truly low resource setting would be one with only a small number of relevant annotations for the task in hand. These can often be found in languages other than English or for more obscure biomedical NER tasks, compared to the comparatively mainstream tasks examined. Further to that, the concept of transfer learning involves training on one different task first and then training on the target task. The prior task in this work is predicting UMLS terms, which is a superset of the different biomedical entities in the eventual target tasks. The results in Table 3 shows this, as the classifiers can predict many of the entities (even with poor F1) with no fine-tuning on the task. So again, this transfer learning primarily works because the prior task is so similar to the final target task. This is an interesting result but should be framed appropriately.

**Response 1:**

We agree with the reviewer that there may be significant overlap between the entity types in our pre-training datasets and the downstream NER test sets. Therefore, our claim that these datasets are “low-resource” because we limit the number of labelled training examples may not be appropriate. To address this, we have made the following changes:

1. We have reframed our main claims to focus on NER applications with limited training samples that focus on common biomedical entities such as those covered in UMLS. We have also adjusted our title to reflect this change. We note that because UMLS covers a very wide range of biomedical entities, we expect that most biomedical NER applications will have some overlap with UMLS, which is why our pre-training datasets are so appropriate.
2. We have added a new Table 3 on page 12 in which we measure the percentage of unique entities in each downstream NER dataset that also appear at least once as labeled entities in the pre-training datasets. We further show in our results section that there is no clear relationship between the precent overlap in entities and how much pre-training improves performance on the downstream NER dataset. Low entity overlap in the pre-training dataset does not necessarily mean that transfer learning will not give a significant performance boost, and vice versa.
3. In our Discussion section (please see pages 22 and 23), we have included an additional set of experiments on TAC SRIE, which we consider a more accurate representation of a low-resource dataset. The TAC SRIE dataset consists of the "Material and Methods" section from 100 toxicology papers, and the entity types of interest are generally not covered within the entity types from UMLS. We further note that we include a sub-experiment in which we test on TAC SRIE without the “species” and “sex” entity types -- we exclude "species" because this entity type is most likely to overlap with UMLS and therefore the pre-training sets, and we exclude "sex” because this entity type is usually a simple keyword search for "male" or "female”. We show that pre-training and self-training can still give performance boosts on this dataset. However, we state that a broader study utilizing a wider range of different types of low-resource NER datasets is required to establish the effectiveness of our methods in low-resource settings.

**Point 2:** Comparing against SemRep (MetaMap) and Scispacy aren’t great baselines. Both of them seem to be predicting UMLS terms so haven’t been trained specifically for the tasks. For a fuller context, we really need better comparisons. You either need to show how other good NER tools behave with such small training sets or use your methods for one of the smaller tasks with the complete dataset + pre-training + self-training and compare it to other tools.

**Response 2:**

While we include SemRep/MetaMap and scispaCy as baseline comparisons due to their popularity, the true baselines are the BiLSTM-CRF and BlueBERT without transfer learning or self-training. We chose these two models because the BiLSTM-CRF is extremely common in NER and because BlueBERT is currently close to state-of-the-art in biomedical NER (the very recent PubmedBERT from Microsoft published this August may be slightly better).

In Table 5 on page 16, we show the baseline performance of these two approaches without pre-training or self-training on 1000 samples from each dataset and show how pre-training can boost performance. In Table 6 (page 17), we provide a similar baseline for these two approaches without self-training and show how self-training can further boost performance on each dataset.

We note that our method of combining pre-training with self-training is model agnostic and, in most cases, should be compatible with any deep learning model. Therefore, our goal is not necessarily to compare with other approaches that do not use pre-training and self-training but to show that our approach can enhance existing NER tools such as the BiLSTM-CRF and BERT (arguably the two most popular deep-learning approaches for NER currently in use).

**Point 3:** The statement about code availability is not reasonable and code should be made available during peer review

**Response 3:**

We apologize, we were still in the process of cleaning our code when we submitted our paper. We have updated our paper to include a link (https://code.ornl.gov/biomedner/biomedner) to our code (page 26).

**Point 4:** Removing statements about low-resource settings, the main conclusions seem to be that transfer learning from UMLS and self-training often gives a boost for biomedical NER, where the entities are a subset of UMLS and in short supply. This is a good contribution and is well-written, but the scope and conclusions need to be reframed accordingly.

**Response 4:**

We agree with the reviewer and hope that our response to Comment 1 has adequately addressed this concern.

Minor
**Point 5:** The mentions of CRISPR & COVID as potential low resource settings seem click-baity. It also doesn’t relate to any of the datasets used and should be removed or be explained and better supported.

**Response 5:**

We agree with the reviewer and have removed all mentions to CRISPR and COVID from our paper because we do not include any experiments directly related to these subjects. Instead, we have included the new set of TAC SRIE experiments in our Discussion section (see Response 1 Point 3).

**Point 6:** The paper makes reference to the SemRep tool for entity extraction. I believe they really mean MetaMap, which is the underlying entity extraction tool. SemRep extracts relations between these entities.

**Response 6:**

Thank you for pointing this out, we have confirmed that SemRep uses the MetaMap tool for NER and have updated our paper to reflect this.

**Point 7:** For the results presented in Table 5, we really need to know what fraction of the complete dataset is being used for Fine Tuning. This would enable clearer comparison with the Fully Supervised set. Are 2000 samples a lot of the dataset for each one or a fraction?

**Response 7:**

We agree with the reviewer that this is important information. Because each of our eight datasets is different in size, for a given dataset, a fixed subset such as 2000 samples is a different fraction of the total dataset. When we constructed our Results tables, we could not include this information without the table becoming too messy. Therefore, we refer readers to Table 2 on page 12, which includes the number of sentences in each dataset, so that they can calculate this information. We have added an additional reference to this Table in our Results section to remind readers that this information is available.

**Point 8:** In Figure 2, “active learning iteration” is a confusing axis label? Perhaps “Iteration of self-training”?

**Response 8:**

We agree with the reviewer that this label is confusing. We have changed this to axis label to “Self-Training Iteration”.

**Point 9:** The paper is very well-written and easy to follow. There are a number of tiny mistakes listed below:
- “we generate pseudo-labels” -> “we generate psuedo-labels”
- “a wide range of naturaly language processing” -> “a wide range of natural language processing”
- “showed that pre-training an BiLSTM-CRF” -> “showed that pre-training a BiLSTM-CRF”
- “labels for NER ragging” -> “labels for NER tagging”
- “SciScpacy” -> “scispaCy”
- “both transer and semi-supervised” -> “both transfer and semi-supervised”

**Response 9:**

Thank you for pointing these out. We have gone through our manuscript and have corrected these and other grammatical errors.
