# Supplementary material for: A pre-training and self-training approach for biomedical named entity recognition
Source: PLoS One. 2021 Feb 9;16(2):e0246310. doi: 10.1371/journal.pone.0246310 (PMC7872256; doi:10.1371/journal.pone.0246310)
Supplement: Attachment — Submitted filename: Plos_NER_rev2.docx [file pone.0246310.s006.docx]

**Reviewer #1**:

In the first review, I had four comments. In this revision, the reviewers addressed those comments.

We thank the reviewer for taking the time to review our paper again.

**Reviewer #2**:

I would like to thank the authors for their substantial revisions and responses to our comments. I feel the paper has been dramatically improved and that the problem reframing seems a lot more suitable. I have a few remaining issues and a few minor things that I hope can be addressed. Overall, I think this is excellent work and has made me think a lot about self-training and methods of transfer learning.

Thank you for the positive feedback, we are hopeful that our work will be useful to the community.

At the moment, the first result (Table 4) reads like an odd experiment. It seems that the hypothesis is that pre-training alone might provide okay performance for problems with no training samples. Could you move that justification (which seems to be in the final sentence of the paragraph discussing Table 4) further up so it's clear why you're showing those results. At first read, I couldn't remember why you were showing results of a model without any fine-tuning which feels weird and had to stop and read around a bit.

Thank you for pointing this out. We have added additional sentences on page 13 to introduce these experiments:

- In our first set of experiments, we evaluate the benefits of pre-training in settings where no labeled training data is available in the target domain.
- In our next set of experiments, we include limited labeled training data from the target domain and then re-evaluate the effects pre-training.

For the MetaMap/scispacy baselines, do you filter for entities of the relevant type for each dataset? They are presumably predicting all UMLS terms and I believe should normalize them to UMLS entities, which could be filtered by type. As an example, do you only include MetaMap predictions of UMLS terms that are of type "gene/protein" in the BC2GM dataset?

We do not filter any entity types for the MetaMap/scispaCy baselines. While MetaMap is capable of predicting entity type along with entity boundaries, the standard scispaCy “en_core_sci_lg” model which we use for our experiments predicts entity boundaries only and does not include functionality for predicting entity type. For consistency, we did not include entity types for either model.

We further note that for some downstream applications such as TAC SRIE, UMLS entity types may not directly match up with the entity types of interest; therefore, filtering entities by UMLS entity type is not possible in these applications.

I wonder why you've put the TAC SRIE result in the Discussion section. It adds nice weight to your argument about NER outside of UMLS. It seems like it would be a nice final result, but really that's up to you.

Thank you for this suggestion. We agree with the reviewer and have moved this into our Results section.

And here are a few small tweaks.

- In related work, I'm not sure I would describe a BiLSTM-CRF as simple. I get that it is comparatively simple compared to a language model like BERT. But there are much simpler NER frameworks, so that probably needs a rephrase.

- Page 15, 18, 21 & Table 5: "Medmentions" -> "MedMentions"

- Page 22, 23, Table 8 caption & Table 10: "Semmed" -> "SemMed"

Thank you for pointing these out. We have addressed all of these.
